# WHO Global Situational Alert System: a mixed methods multistage approach to identify country-level COVID-19 alerts

**DOI:** 10.1136/bmjgh-2023-012241

**Published:** 2023-07-26

**Authors:** Martina McMenamin, Jessica Kolmer, Irena Djordjevic, Finlay Campbell, Henry Laurenson-Schafer, Jessica Lee Abbate, Basma Mostafa Abdelgawad, Amarnath Babu, Thierno Balde, Neale Batra, Victoria D Bélorgeot, Hannah Brindle, Tshewang Dorji, Marjam Esmail, Ingrid Hammermeister Nezu, Lucía Hernández-García, Mahmoud Hassan, Friday Idoko, Sarah Karmin, Zyleen A Kassamali, Masaya Kato, Tamano Matsui, Mengjuan Duan, Villyen Motaze, Opeayo Ogundiran, Boris I Pavlin, Ana Riviere-Cinnamond, Kathleen Ryan, Tanja Schmidt, Tika Sedai, Maria D Van Kerkhove, Teresa Zakaria, Michael Höhle, Abdi R Mahamud, Olivier le Polain de Waroux, WHO Global Situational Alert System Group, Dominic Cocciolone

**Affiliations:** 1 WHO Health Emergencies Programme, WHO Headquarters, Geneva, Switzerland; 2 WHO Health Emergencies Programme, WHO Regional Office for Africa, Brazzaville, Republic of Congo; 3 WHO Health Emergencies Programme, WHO Regional Office for the Eastern Mediterranean, Cairo, Egypt; 4 WHO Health Emergencies Programme, WHO Regional Office for South-East Asia, New Delhi, Delhi, India; 5 WHO Health Emergencies Programme, WHO Regional Office for the Americas, Washington, DC, USA; 6 UNICEF, Geneva, Switzerland; 7 WHO Health Emergencies Programme, WHO Regional Office for Europe, Copenhagen, Denmark; 8 WHO Health Emergencies Programme, WHO Regional Office for the Western Pacific, Manila, Philippines; 9 Stockholm University, Stockholm, Sweden

**Keywords:** COVID-19, Public Health, Epidemiology

## Abstract

**Background:**

Globally, since 1 January 2020 and as of 24 January 2023, there have been over 664 million cases of COVID-19 and over 6.7 million deaths reported to WHO. WHO developed an evidence-based alert system, assessing public health risk on a weekly basis in 237 countries, territories and areas from May 2021 to June 2022. This aimed to facilitate the early identification of situations where healthcare capacity may become overstretched.

**Methods:**

The process involved a three-stage mixed methods approach. In the first stage, future deaths were predicted from the time series of reported cases and deaths to produce an initial alert level. In the second stage, this alert level was adjusted by incorporating a range of contextual indicators and accounting for the quality of information available using a Bayes classifier. In the third stage, countries with an alert level of ‘High’ or above were added to an operational watchlist and assistance was deployed as needed.

**Results:**

Since June 2021, the system has supported the release of more than US$27 million from WHO emergency funding, over 450 000 rapid antigen diagnostic testing kits and over 6000 oxygen concentrators. Retrospective evaluation indicated that the first two stages were needed to maximise sensitivity, where 44% (IQR 29%–67%) of weekly watchlist alerts would not have been identified using only reported cases and deaths. The alerts were timely and valid in most cases; however, this could only be assessed on a non-representative sample of countries with hospitalisation data available.

**Conclusions:**

The system provided a standardised approach to monitor the pandemic at the country level by incorporating all available data on epidemiological analytics and contextual assessments. While this system was developed for COVID-19, a similar system could be used for future outbreaks and emergencies, with necessary adjustments to parameters and indicators.

**WHAT IS ALREADY KNOWN ON THIS TOPIC**

- Surveillance data vary substantially across countries and regions, and over time, making standardisation of risk assessments in a global emergency challenging.
- Aside from the time series of epidemiological data, information from public health intelligence is often available that would enhance the situational assessment and enable a more coordinated and effective response; however, it is not straightforward how to combine these data for a more unified process, or how to account for subnational-level data which is an additional challenge in a limited resource setting.

**WHAT THIS STUDY ADDS**

- This study presents a flexible and systematic approach to incorporate several sources of information of varying robustness for public health situational analyses.
- The process described allows for uncertainty at each stage to be carried through to the decision for the final alert level.
- In addition to methodology, the work describes an example of implementation at a global level during a health emergency and demonstrates the need for collaboration across global, regional and national levels.
- Key operational challenges are discussed to improve future preparedness and response activities, including difficulties in assessing disease burden where surveillance may be poor, such as in humanitarian emergencies, as well as political sensitivities related to reporting alert levels.

**HOW THIS STUDY MIGHT AFFECT RESEARCH, PRACTICE OR POLICY**

- The mixed methods approach outlined in this manuscript could be used in future health or other emergencies as a systematic way to integrate multiple data sources of varying quality to facilitate risk assessment and decision-making.
- The lessons learnt from the implementation of the yearlong process across global, regional and national levels during the COVID-19 pandemic will inform and refine future applications of such a process.

## Background

Globally, since 1 January 2020 and as of 24 January 2023, there have been over 664 million confirmed cases of COVID-19 and over 6.7 million deaths reported to WHO.^1^ The COVID-19 pandemic has been marked by periods of increased transmission, with surges occurring at different times across the globe. In an effort to better prepare for, identify and ultimately respond to these epidemic waves, WHO developed the Global Situational Alert System (GSAS) for COVID-19, a public health alert system using a mixed methods approach to support the identification of countries or territories for which immediate actions may help to mitigate the impact of a surge in COVID-19 morbidity and mortality.

The alert system was developed in early May 2021, following the rapid deterioration of the COVID-19 situation in India and Nepal. During this time, national authorities, United Nations agencies, non-governmental organisations and other response entities faced significant challenges mobilising resources due to the sharp increase in infections, hospitalisations and deaths over a short period, combined with an underprepared healthcare system and further compounded by global supply constraints and workforce limitations.^2 3^ The primary objective of the alert system was to enable the early identification of similar situations where healthcare capacity may become overstretched due to a surge in COVID-19 morbidity and mortality, or where other contextual factors may aggravate COVID-19 transmission and impair response capacities. These factors include issues such as supply shortages, mass gathering events, concurrent outbreaks and instability or insecurity related to acute events. The outputs were used as a tool to inform, guide and streamline coordination, operational and technical support and the advance allocation of critical resources. The process provided a global mechanism to enable teams at WHO headquarters and regional offices to regularly assess the COVID-19 situation within each country using all available information on epidemiological and contextual factors. Differences in surveillance systems and their completeness were also accounted for to provide a standardised approach in which all countries and regions could be proactively offered support when a situational alert was raised. The process also enabled WHO and partners, including UNICEF, to develop and share guidance on the importance of maintaining other essential services, including access to education.

The objectives of this paper were twofold: (1) to provide a description of the methodology for the multistage GSAS, and (2) to present an evaluation of the process, including a country case example, to demonstrate the resulting operational response. We reflect on challenges from developing and implementing such a system in an ongoing pandemic and highlight considerations for adaptation and use for future outbreaks and pandemics, as well as other types of emergencies.

## Methods

### Description of the mixed methods approach

**Figure 1:**
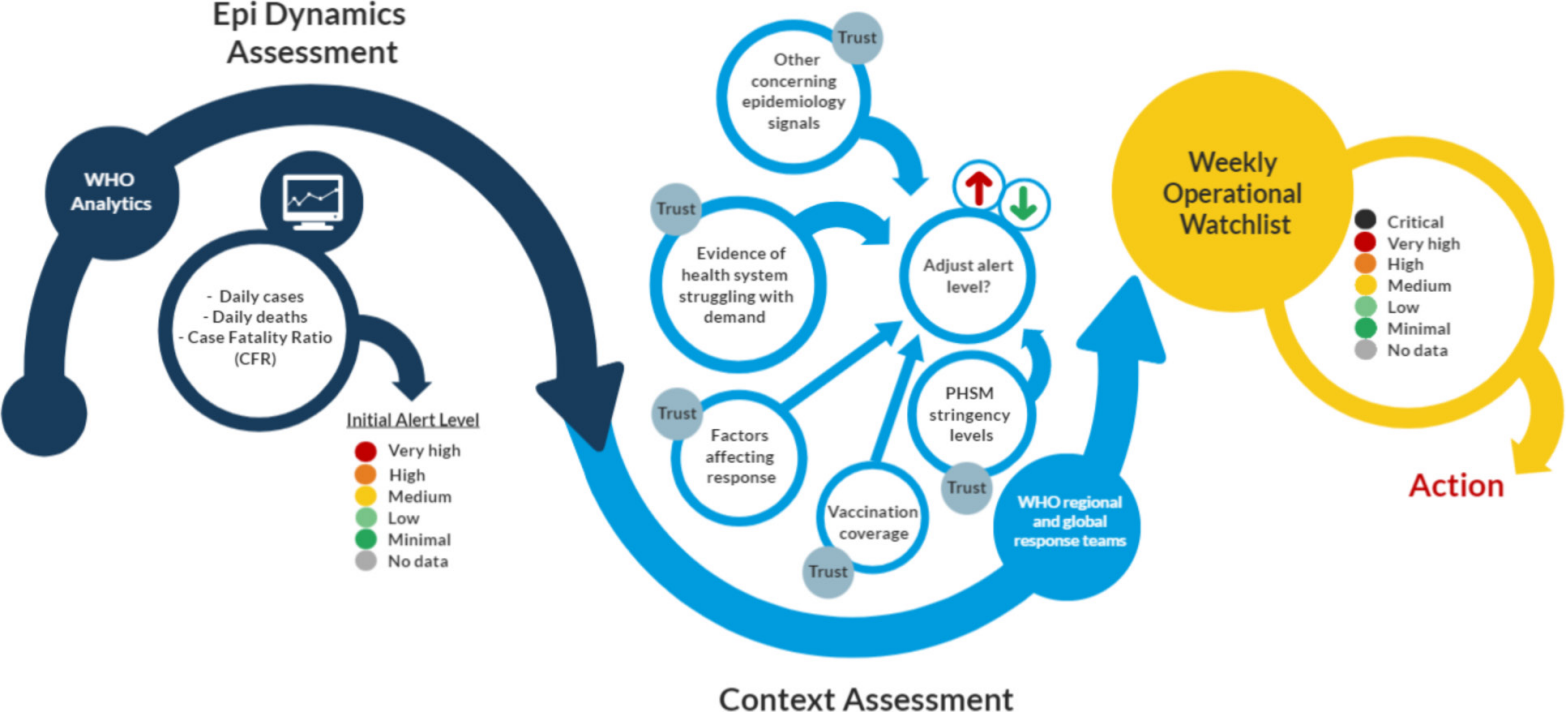
Multistage process for the weekly Global Situational Alert System (GSAS). PHSM, public health and social measure.

The WHO GSAS for COVID-19 comprised a three-stage methodology (figure 1), with assessments conducted on a weekly basis for 237 countries, territories and areas starting from May 2021. The methodology and weekly functioning of the system evolved throughout the period of implementation, where the underlying algorithms and operational approaches were regularly amended to meet the changing global state of the pandemic. In what follows, we describe the most recent version of the system implemented in the second half of 2021.

#### Stage 1: epidemiological dynamics algorithm

The automated statistical risk assessment algorithm which produced the initial dynamics alert level was based on the time series of cases and deaths as reported to WHO for each country. The metric of interest was the predicted total number of ‘true’ COVID-19-associated deaths within the next 5 weeks per 1 million population, obtained by correcting the projected reported figures using an adjustment factor. The fatality rate was considered to be the best proxy for severity at the global level at that point in time, in the absence of consistent data on hospitalisation and intensive care unit (ICU) admission from all countries. The alert levels were then obtained by thresholding this quantity into five classes: ‘Very High’, ‘High’, ‘Medium’, ‘Low’ and ‘Minimal’ (see online supplemental appendix 1 for more details). Future deaths were predicted from the time series of reported cases and from estimating a real-time country-specific reported case fatality ratio. Furthermore, future reported deaths were made more comparable between regions using adjustment factors based on WHO country-specific excess mortality estimates.^4^ These estimates were grouped by the World Bank income groups (high income, upper middle income, lower middle income and low income) to form four adjustment factor distributions to be applied to the reported death time series.^5^ For a number of countries (n=42), this approach was further augmented by setting the adjustment factor manually after consulting expert opinion from country and regional offices, based on knowledge about how fatality rates in particular countries translated into hospitalisation and public health burden. Uncertainty in both the future cases and the adjustment factor were incorporated into the overall uncertainty in the projected deaths. This stage was automated but enhanced with some manual checks on the alert level outputs to identify any instances where the algorithm had failed. More information on the methods is available in online supplemental appendix 2.

10.1136/bmjgh-2023-012241.supp1 Supplementary data

10.1136/bmjgh-2023-012241.supp2 Supplementary data

#### Stage 2: context assessment

The second stage involved the inclusion of contextual information for each country to complement the data obtained from indicator-based surveillance. Contextual factors were manually assessed using signals for each country based on three indicators: (1) pressure on the healthcare system; (2) other concerning epidemiological signals (eg, changes in test positivity rates); and (3) factors affecting the response (eg, mass gatherings, natural disasters, civil unrest, armed conflict or humanitarian displacement), which may impact the functioning of surveillance systems, and capacity to implement public health and social measures (PHSMs). Qualitative information was gathered from public health intelligence including WHO country and regional office situation reports, Ministry of Health websites and the WHO SARS-CoV-2 variant tracking database,^6^ as well as additional intelligence from the field on capacity of health systems and health workers provided by UNICEF. This was subsequently used to inform the risk level selected for each indicator (online supplemental appendix 3, online supplemental appendix S1). Trust levels were used to augment these indicator levels to capture uncertainty in the assessment resulting from differing amounts and quality of information available between countries and weeks. Indicator levels and associated trust levels of these manually updated indicators were combined with automatically updated information on three additional indicators: vaccination coverage, the stringency of PHSMs and whether the country is experiencing a humanitarian emergency (defined as countries affected by large humanitarian emergencies for which there is a consolidated multiagency Humanitarian Response Plan).^7^ This produced a recommendation on whether a country should be maintained at the initial dynamics alert level, or whether this level should be increased or decreased (online supplemental appendix 4). An additional alert level of ‘Critical’ was possible at this stage for countries deemed as ‘Very High’ during the first stage and subsequently recommended for an increase in alert level at stage 2.

10.1136/bmjgh-2023-012241.supp3 Supplementary data

10.1136/bmjgh-2023-012241.supp4 Supplementary data

The contextual assessment stage was implemented within a Bayes classifier framework,^8^ where points were assigned to each indicator level, and point thresholds were set for upgrading or downgrading the initial alert level. These were initially selected based on user elicitation and later estimated from the available data to balance the sensitivity and specificity of the system, with priority given to the sensitivity so as not to miss alerts. To account for the fact that the information used for the contextual assessment was very heterogeneous, the points were further augmented using a trust level to characterise uncertainty in the assigned points. The associated trust level for each indicator determined the distribution over the possible indicator levels, where a ‘High’ level of trust in the available information resulted in adding the points for the selected level directly to the overall risk score (ie, variance zero), and lower trust levels gave weight to the other indicator levels (ie, variance in how many points were assigned, online supplemental appendix 4). This approach was designed and implemented to standardise the decision-making process for public health professionals assessing the situational level using existing signals, while still maintaining the qualitative nature of the contextual assessment. Using this procedure, teams at the WHO global and regional levels, including UNICEF colleagues, assessed the algorithm and contextual assessment outputs on a weekly basis and jointly agreed on a final alert level for each country. This was achieved by either accepting the recommendation from the classifier or by over-riding this suggestion for reasons that were subsequently documented. This stage included considerations provided by experts at WHO regional offices for informal or subnational data to decide the final alert level.

#### Stage 3: response

A weekly operational watchlist of country alerts to monitor was produced and included countries assessed to have High, Very High and Critical final alert levels. Since the initiation of the alert system in May 2021, the weekly analysis was used to support a shared understanding of risk and operational priorities at a global and regional level, including partner organisations. This was accomplished through the weekly interaction between global and regional teams to develop the watchlist as well as weekly engagement between COVID-19 incident managers to conduct horizon scanning,^9^ flag critical areas for support, share technical expertise and reflect on lessons learnt in response to at-risk contexts. We illustrate the approach using the country case study of Romania during the wave caused by the Delta variant of concern (VOC) in 2021, which details how the system used early identification for efficient response coordination.

### Data sources

The WHO GSAS used several data sources for the evaluation of epidemiological dynamics and context assessment including case and death time series reported to WHO, vaccination coverage, WHO PHSM index and the Epidemic Intelligence from Open Sources database.^1 7 10–14^ More details on the data sources used at each stage of the process are included in online supplemental appendix 3.

### Evaluation

Throughout the process, continuous evaluation of the system based on expert elicitation, feedback from end users and data-driven assessments informed tweaks of parameters and processes, as well as more substantial changes to the system. Between May and June 2022, a retrospective quantitative review of the process was undertaken to inform the use of the system for the COVID-19 response and consolidate lessons learnt to make use of similar systems in future outbreaks and other health emergencies. The assessment period covered July 2021 until June 2022, which corresponds to global circulation and dominance of the Delta (July to December 2021) and Omicron (January to June 2022) VOCs, as determined by sequences reported to the Global Initiative on Sharing All Influenza Data.^15 16^ Using the data on weekly alert levels for 237 countries, territories and areas between assessment weeks beginning on 5 July 2021 and 13 June 2022, we illustrate and evaluate the system in a number of key areas: (1) we identify the components of the process responsible for final alerts on a weekly basis; (2) we evaluate the predictive performance of the system in terms of accuracy using metrics internal to the process, and assess validity using hospitalisation and ICU data from Our World in Data^17^; and (3) we assess the timeliness of the alert system in relation to surges in reported cases and deaths. To assess the components responsible for alerts, we assess the proportion of final alerts identified at the first stage of the algorithm for each week assessed and also present absolute numbers by WHO region. To evaluate timeliness, we identify the largest peak in reported cases and reported deaths for each country and, for those that progressed to being listed on the operational watchlist because of that wave, we determine how many weeks they were on the watchlist before the peak in reported cases and reported deaths. We assess the validity according to hospitalisation and ICU data both visually and using Kendall’s coefficient of rank correlation to assess agreements between the alert level and hospitalisation numbers.

Feedback from contributors and end users was obtained via a two-step process conducted in March 2022. First, an anonymised electronic survey was used to collect feedback using the ‘LimeSurvey’ tool, followed by semistructured group discussions to focus on survey results and obtain additional insights into recommendations for future applications. The qualitative data from the group discussions were manually reviewed and organised into themes by two rapporteurs. The operational evaluation focused on the following key areas: (1) usefulness and operational relevance of the system, (2) extent to which the system was fit for purpose and (3) improvement for future adaptations of the system.

### Patient and public involvement

Patients or members of the public were not involved in the design, conduct or reporting of this research.

## Results

### Summary of global alert trends

Between July 2021 and June 2022, the largest number of countries on the global operational watchlist in each week occurred near the beginning of the ‘Omicron wave’ in mid-January 2022, with the number of countries globally identified as either ‘Critical’, ‘Very High’ or ‘High’ beginning to decline thereafter. Periods where the highest alert levels (Critical, Very High) were raised varied between regions: WHO’s South-East Asia Region had the highest proportion of countries with Critical or Very High alerts in mid-2021, while in WHO’s European Region this occurred in early 2022 (online supplemental appendix 5 figure S1). Only 12 countries reporting data did not have alerts raised at some time between July 2021 and June 2022, of which 10 were small island nations in the Western Pacific Region and two were countries in the Eastern Mediterranean Region, which had few or no reported cases during this time.

10.1136/bmjgh-2023-012241.supp5 Supplementary data

### Algorithm components responsible for alerts

**Figure 2:**
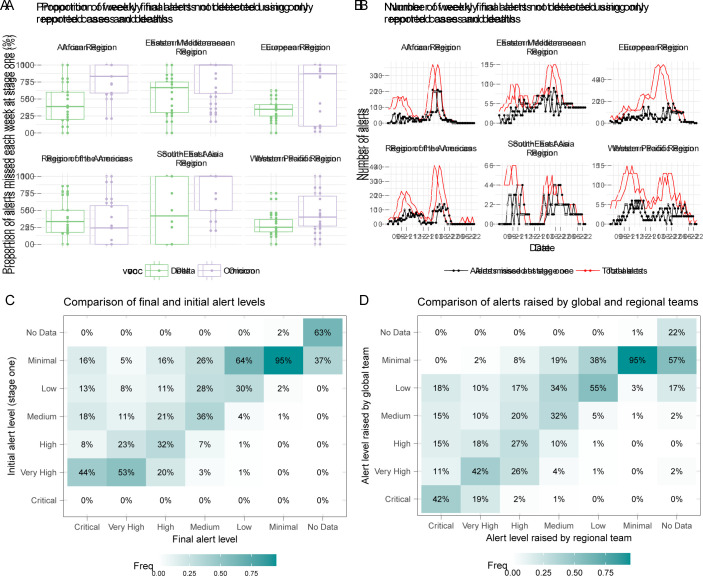
Summary of algorithm components responsible for alerts and the proportion of weekly final alerts identified using contextual information, by region and variant of concern (VOC). (A) Number of alerts missed using only reported cases and deaths (B). The Delta period incorporates assessments from July to December 2021 and the Omicron period incorporates assessments from January to May 2022. (C, D) The comparison is only for the weekly alerts between 3 January 2022 and 18 April 2022, when the process incorporated the standardised context assessment. (C) Note that a ‘Critical’ alert level could not be raised, by definition, at stage 1.

Globally, 44% (IQR 29%–66%) of weekly ‘Critical’, ‘Very High’ and ‘High’ alerts raised each week between July 2021 and June 2022 would not have been identified using reported cases and deaths alone. This is true across both the Delta and Omicron VOC dominant periods at a WHO regional level, with a higher proportion of alerts each week identified at the first stage during the Delta period (figure 2A). However, this depends on the absolute number of alerts each week, which varied throughout the assessment period and across WHO regions (figure 2B). In most cases, the contextual assessment raised the initial alert level rather than reduced it (figure 2C), and we observed situations where additional alerts were flagged using more detailed information provided by teams at the WHO regional level, or by UNICEF colleagues, which may have otherwise been missed using only the information available at a global level (figure 2D). Where discrepancies occurred at the final stage, the higher alert level was typically selected as per the preference for higher sensitivity of the system and to account for potential reporting delays.

### Validation and predictive performance

#### Internal metrics

**Figure 3:**
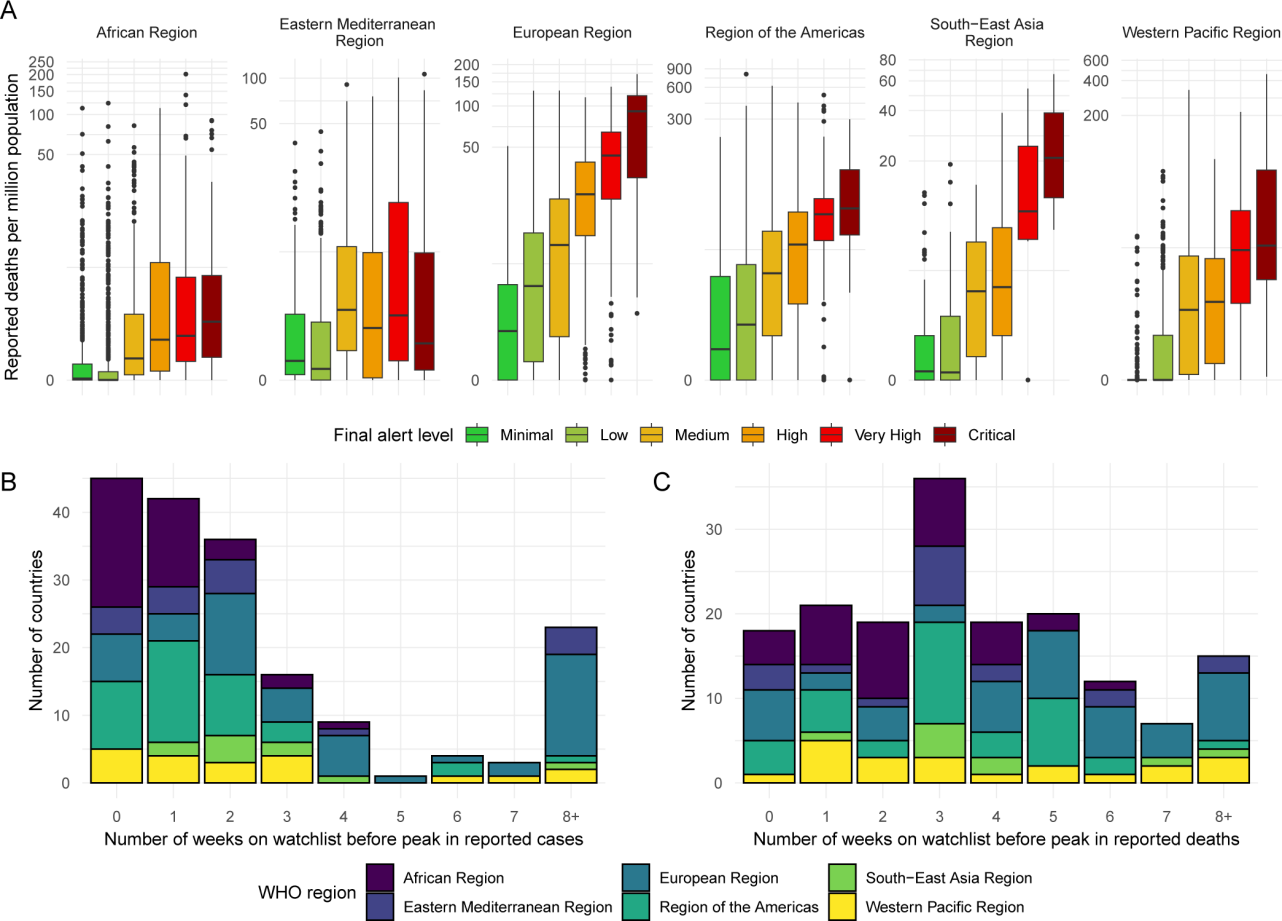
(A) Reported deaths per million population across alert levels and WHO regions. Note that reported deaths are shown on a log scale. (B) Timeliness of identifying countries to include on the watchlist before the reported peak in cases, shown by WHO region. (C) Timeliness of identifying countries to include on the watchlist before the reported peak in deaths, shown by WHO region.

Comparing the distribution of reported deaths across alert levels and regions, we generally observe an increasing trend in average deaths per million population, with some overlap between levels (figure 3A). The thresholds for alert levels with respect to reported deaths vary across regions; however, this is expected due to variation in estimated under-reporting of deaths, which is accounted for in the algorithm. In countries with humanitarian emergencies, this under-reporting was estimated to be higher, with no reporting at all in some cases. The timeliness of the alerts varied with respect to WHO region, where many countries in the European Region were identified more than 2 months prior to their peak in reported cases while many countries in the African Region were only identified 0–2 weeks prior to their peak in reported cases (figure 3B). We observe improved timeliness of alerts with respect to reported deaths, as this is a lagged indicator, with only 17 countries not included on the watchlist prior to their peak in reported deaths across the period assessed (figure 3C).

#### External metrics

**Figure 4:**
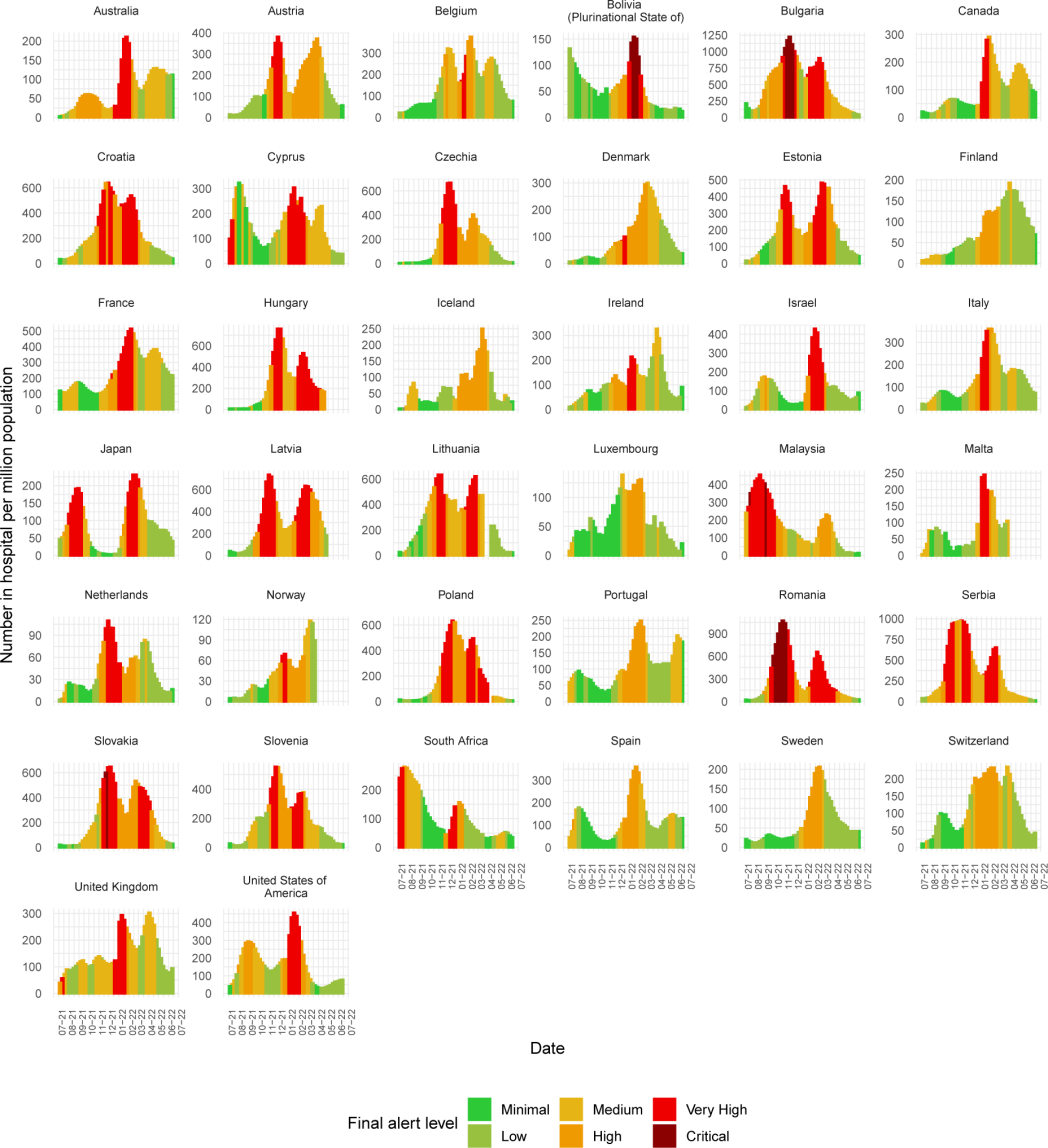
Trends in numbers of cases in hospital per million population versus the situational alert level for a subset of countries in the European Region, the Region of the Americas and the Western Pacific Region for which this information is available. External data source: Our World in Data. Note that the hospitalisation data (availability and quality) vary among WHO member states based on a variety of factors and the data above are not representative of all member states.

Alert level trends generally tracked the reported numbers of cases in hospital well (figure 4), and this was also true for numbers in ICU (online supplemental appendix 5, figure S2). The rank correlation between weekly hospitalisations per capita and weekly alert level is 0.52 (95% CI 0.49, 0.54); and between weekly number in ICU per capita and weekly alert level is 0.35 (95% CI 0.32, 0.38). From the subset of countries assessed, the alerts are valid in identifying times of increased pressure on hospital capacity and were flagged in advance of the peak in hospitalisations in most cases (figure 4). However, Luxembourg was only added to the watchlist when hospital numbers had nearly peaked. Furthermore, for the first peak in hospitalisations in Cyprus and Finland, alert levels were reduced as hospitalisations were still rising; however, this could also be due to a possible change in capacity within countries during this time. For ICU occupancy, we also observe large peaks in Argentina and Chile in July 2021 that were not reflected in the situational alert level; however, hospitalisations were already decreasing at this time and the algorithm was still in the early stages of development (online supplemental appendix 5, figure S2). Alerts raised for Belgium in late 2021 lagged ICU demand, as the alert level continued to rise even though ICU admission rates were reported to be declining.

Using the distribution of numbers of cases in hospital and numbers in ICU per million population across alert levels in the subset of countries with available hospitalisation data, we observe that the alert level trend increases with increasing hospitalisation and ICU admission rates, indicating satisfactory validity of final alert levels for this subset of countries cumulatively over the assessment period, despite some overlap between levels (online supplemental appendix 5, figure S2). The agreement between alert level and hospitalisation is higher than that of ICU admissions, indicating the system was more accurate in predicting hospitalisation than ICU demand. This is also reflected in the rank metric.

### Country case study: Romania

To demonstrate the operational benefits of the system, we present the case study of Romania during the Delta wave in late 2021. This example highlights where early identification prompted rapid mobilisation across all pillars of response and led to coordinated support. In addition, it demonstrates where data officially submitted to WHO were augmented with external reports on hospital occupancy and patient transfers to inform the situational assessment. However, it is important to note that this is not representative of all instances of operational support. In some instances, WHO support was provided through technical advice, support with operational planning and advocacy rather than the deployment of human resources and supplies. In other instances, WHO was not able to provide support due to limitations in supply chain, access or other.

**Figure 5:**
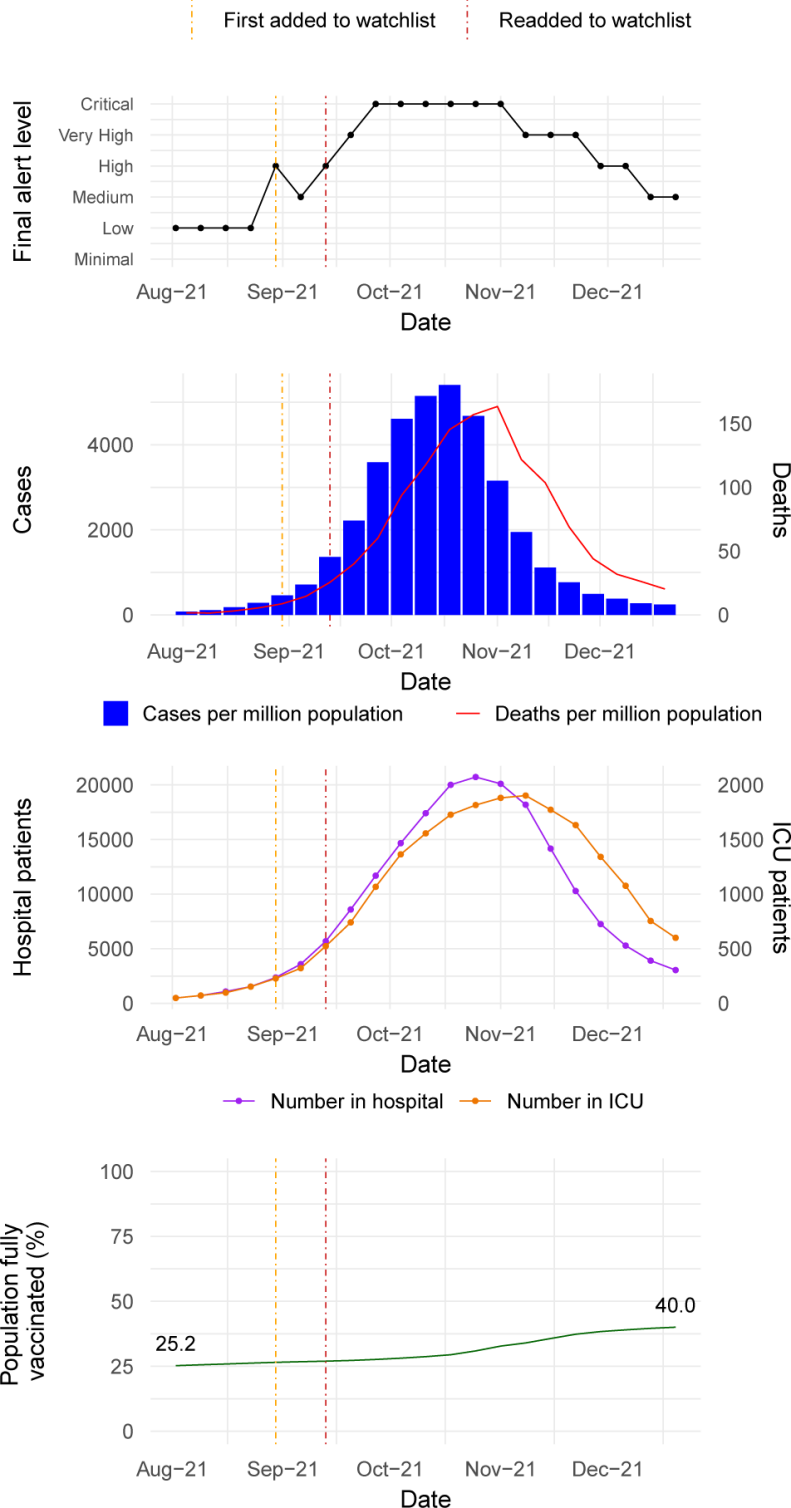
Summary of trends in final alert levels, cases and deaths, hospitalisation and intensive care unit (ICU) numbers and vaccination coverage for Romania during the ‘Delta wave’ between August and December 2021.

Between August and December 2021, Romania experienced its third wave of COVID-19 cases due to the Delta VOC, peaking at over 100 000 new confirmed cases from 18 to 24 October 2021. As of 1 October 2021, only 28% of the total population had received the primary series of vaccination, the healthcare system was overwhelmed and there were reports of patient transfers abroad.^18^ From early September 2021, the alert level was upgraded by one step each week and on 4 October 2021, Romania was classified at the highest alert level, ‘Critical’. A ‘High’ alert level had been assigned to Romania 7 weeks prior to the peak in confirmed case numbers and 9 weeks prior to peak hospital occupancy (figure 5).

As a result of the alert, WHO deployed an incident manager for COVID-19 to Romania in mid-October 2021 and a high-level mission was undertaken by the WHO Regional Emergency Director for the European Region and the Executive Director of the National Institute of Public Health of Romania. Supplies including rapid diagnostic tests, personal protective equipment (PPE) and oxygen were rapidly deployed and the country was identified as a priority for risk communication and community engagement and a tailored messaging and communications strategy was developed. Epidemiological modelling was undertaken in collaboration with the National Institute of Public Health in Romania. The COVID-19 PHSM calibration tool^13^ developed and implemented by the WHO European Region was used regularly to generate situationally appropriate guidance on interventions; and vaccination data were used for action assessments and to prompt a follow-up mission on vaccine uptake. The actions were coordinated with the European Centre for Disease Prevention and Control to ensure the approaches were aligned and to leverage joint capacity.

During the second week of November, the alert level was downgraded to ‘Very High’ and did not reach a ‘Medium’ level until mid-December. During the de-escalation period, despite a decrease in cases and deaths resulting in an initial dynamics alert level of High, pressure on the healthcare system identified during the contextual assessment led to the decision to maintain a final alert level of ‘Very High’ for 3 weeks, demonstrating the prioritisation of sensitivity as a key feature of the system.

## Discussion

The WHO GSAS for COVID-19 provided a systematic approach to combine epidemiological information with a contextual assessment to allow for additional insight into the extent of global outbreaks at a country level in 237 countries, territories and areas on a weekly basis between May 2021 and June 2022. While the global system was paused in June 2022, some WHO regions adapted the methodology and continued to run regional assessments. The output, in the form of a global operational watchlist, informed and supported resource allocation, advocacy, funding release and critical assistance. The system supported the release of more than US$27 million from WHO emergency funding to help expedite response activities in at-risk contexts based on the generated watchlists, namely countries in the South-East Asian Region and the African Region. The weekly analysis and accompanying operational watchlist also informed the rapid release of operational and technical support, including over 450 000 rapid antigen diagnostic testing kits (Ag-RDT) for COVID-19, over 6000 oxygen concentrators, support to deploy and establish COVID-19 treatment centres and deployment of rapid response teams across WHO regions. Beyond the deployment of specific commodities, the results of the weekly watchlist were integrated with supply forecasting activities to help avoid future internal stock-outs of critical supplies and equipment and aided prioritisation. In some WHO regions, the weekly watchlist was also used as a high-level advocacy tool with member states, donors and key partners on resource needs and the importance of specific PHSMs. Internally, incident management teams within WHO regional offices and headquarters used the watchlist to guide and prioritise the work of technical teams and support countries.

From a methodological perspective, the evaluation showed the system performed well according to validation metrics and provided timely alerts for deteriorating situations in most cases. However, the system could have benefited from the automated use of additional data, such as age disaggregated vaccination coverage or hospital bed occupancy, which were not systematically available for all countries. Any data available on these metrics for a given country were manually accounted for in the context assessment, and both the quantitative results and internal user feedback highlighted that both stages of the weekly process were therefore necessary to maximise sensitivity in flagging alerts. Likewise, although reported cases and deaths were chosen as the metric in the statistical algorithm, it was recognised that disease burden is multifaceted and other indicators may be considered to more readily capture this, such as hospitalisations or ICU admissions. However, this information was not available for most countries in real time, and so reported mortality accounting for under-reporting was used to reflect the global nature of the alert system, while predicting mortality from reported cases allowed more timely flagging of future healthcare burden. Furthermore, while data on reported cases and deaths were available for most countries, differences in testing strategies and capacities, as well as COVID-19 case and death definitions used, varied substantially between countries and over time which made the standardisation of alerts at a global level challenging. For the subset of countries with hospitalisation and ICU data available, we assessed the validity of the alert system against these metrics and found the trajectory of alerts to track the trends in hospital and ICU numbers in most cases. However, countries with these data available, even retrospectively, are not globally representative due to the high quality of their surveillance systems, limiting generalisability. In addition, per capita hospital and ICU occupancy versus the situational alert level is not comparable between countries nor over time as it depends heavily on changing capacity and admission processes and does not necessarily account for cases where SARS-CoV-2 is an incidental finding on admission. Furthermore, it is important to note that the results of the quantitative evaluation and feedback from users illustrated some important differences across regions in the performance of the system. In particular, we see that alerts were on average timelier in regions with more rich and frequently reported data on cases, deaths and hospital capacity, such as the European Region. Furthermore, we observe variation in which components of the algorithm were responsible for raising alerts. We see in the Region of the Americas and the Western Pacific Region that stage 1, which uses only the reported cases and deaths, performs better than in the Eastern Mediterranean Region or African Region which are more dependent on the additional data sources used in the context assessment.

Despite these challenges, the flexibility of the mixed methods approach facilitated the evaluation of COVID-19 situations in countries where surveillance and response capacities were impaired or had remained insufficient, and where reported cases and deaths could not reflect the true scale of the pandemic while allowing for uncertainty in varying data sources to be captured. From the operational perspective of implementing and coordinating the process, it was clear that although standardisation is an important aspect of risk assessment at the global level, sufficient flexibility had to be integrated within the overall approach to accommodate this variation, and to ensure outputs of the process were operationally relevant. Therefore, although the system provided structure, the alert system remained subjective to an extent and was strongly affected by risk perception within and across teams, as well as the stage of the pandemic and other factors within the country at that time. Other practical challenges arose from performing the assessment at a country level for larger countries, for which the situation was often heterogeneous, with a lack of subnational data to target the preparedness and response. This was also the case for smaller island nations, where capacity could rapidly become overwhelmed. This resulted in many cases where regional-level understanding was often preferred when deciding a final alert level at a country level and additional country-level understanding would have been beneficial. While these comparisons across countries and regions were challenging overall, the evaluation indicated that the process met the aim of facilitating frequent and detailed assessments of situational alert levels and provided a mechanism for allocation of wider support when needed.

In terms of response activities, developing a shared understanding of public health risk at a global level proved extremely valuable both in terms of encouraging a standardised and collaborative approach to assessing risk, and further enabling multidisciplinary collaboration across the levels of the organisation, as well as within UNICEF who used the system to alert regions and countries and to help prioritise actions for preparedness and response beyond healthcare. While the need for the rapid deployment of essential commodities such as diagnostic tests, PPE and biomedical supplies waned throughout 2021, due to supply chain constraints easing and countries becoming better prepared for future surges in cases, response teams at a global and regional level continued to use the weekly watchlist to inform high-level advocacy and to focus the work of technical and operational teams. However, the retrospective review of the process highlighted that while the system was deemed useful throughout the pandemic, it did not consistently fulfil the operational objective (ie, the prioritisation and offer of technical assistance, supplies, advocacy, etc) to the same extent in all regions. In addition, advance allocation of critical resources was not possible in some instances, due to a short window to respond before a surge would overwhelm capacity, especially in low and middle-income countries. Furthermore, there were political sensitivities around the classification of countries based on the alert levels, which proved in some cases too difficult to navigate and restricted the external use of this system with various partner organisations and with some health ministries.

Future applications of the system could explore how the methodology and process can be refined to better account for limitations in data availability and differences in surveillance architecture. A possible improvement in future iterations would be to tailor the system at a regional level to allow for additional information to be automated in some regions and augmenting the parameter space with expert elicitation in regions or countries where less data are available. Another adaptation could be the inclusion of additional contextual factors, such as changes in healthcare access, as stand-alone indicators rather than all contextual factors being grouped into three indicators. However, achieving sufficient granularity should be balanced with the limited capacity of teams to complete the assessment, which was at times an operational challenge. An adapted system is currently being developed to prioritise country support for the global response to the resurgence of cholera across multiple regions and countries using a combination of the epidemiological situation, an analysis of the public health response capacity and an understanding of contextual factors. The lessons learnt from the development and application of the system during the COVID-19 pandemic are being considered in designing this approach and will be used in future health emergencies.

## Conclusion

The WHO GSAS for COVID-19 provided a systematic approach to monitor the pandemic at the country level by combining epidemiological analytics and contextual assessments and was used to inform the global, regional and national public health response on a weekly basis. The methodology presented here serves as a model that could be applied by countries to make a subnational system of alerts for within-country prioritisation and early detection of situations of concern by using multiple data sources including qualitative assessments. While this system was developed for COVID-19, it could be used for future outbreaks and emergencies, with the caveat of necessary adjustments to parameters and indicators, and a need to encourage capacity building within countries for better and more timely data collection and reporting.

## Data Availability

Data are available in a public, open access repository. Data are available on reported cases, deaths, vaccination and PHSM over time, as referenced in the manuscript.
