# Supplementary material for: WHO Global Situational Alert System: a mixed methods multistage approach to identify country-level COVID-19 alerts
Source: BMJ Glob Health. 2023 Jul 26;8(7):e012241. doi: 10.1136/bmjgh-2023-012241 (PMC10373705; doi:10.1136/bmjgh-2023-012241)
Supplement: Supplementary data [file bmjgh-2023-012241supp001.pdf]

WHO Global Situational Alert System: a mixed methods multistage approach to identify country-level COVID-19 alerts

Appendix 1:

Alert level definitions

Table S1 Interpretation of final alert levels and associated action

Alert level	Situational description	Action
Critical	Extremely unstable situation within country with concerns that many patients cannot access healthcare services either due to extremely large demand or some additional humanitarian instability affecting healthcare services.	Add to operational watchlist, close coordination with the regional office on immediate support needs and monitor closely.
Very High	Healthcare systems are becoming overwhelmed in multiple areas sub-nationally or at a national level and almost all non-COVID-19 related provisions may become affected. Alert levels may also be selected for countries where the system is not yet becoming overwhelmed but additional contextual factors exist that may affect response efforts.	Add to operational watchlist, close coordination with the regional office on immediate support needs and monitor closely.
High	High levels of community transmission with large demands on healthcare services. Hospitals have capacity to treat COVID-19 patients, but other healthcare provisions may be becoming affected or concerning contextual factors have been identified which may impact response efforts.	Add to operational watchlist, discussions on possible support needs, and monitor closely.
Medium	Ongoing community transmission of COVID-19 with low to moderate hospital occupancy. The situation is stable and healthcare capacity can handle demand at a national and subnational level.	Continue to monitor the situation in coordination with the regional office.
Low	Ongoing community transmission of COVID-19 with little impact on the healthcare system.	No action required.
Minimal	Little or no community transmission of COVID-19 with no concerning contextual factors present.	No action required.
No Data	No data available	No action required.
