# Supplementary material for: WHO Global Situational Alert System: a mixed methods multistage approach to identify country-level COVID-19 alerts
Source: BMJ Glob Health. 2023 Jul 26;8(7):e012241. doi: 10.1136/bmjgh-2023-012241 (PMC10373705; doi:10.1136/bmjgh-2023-012241)
Supplement: Supplementary data [file bmjgh-2023-012241supp002.pdf]

WHO Global Situational Alert System: a mixed methods multistage approach to identify country-level COVID-19 alerts

Appendix 2: Statistical Methodology of the Epidemiological Dynamics Classification Algorithm

1.1. Dynamics Alert Algorithm

The algorithm focuses on the *true* COVID-19 associated deaths as a metric for measuring the disease burden of COVID-19 in each country. This is a simplification of a complex multi-faceted situation, ranging from overwhelmed healthcare causing problems in non-COVID related treatments, non-lethal quality of live deprecation (such as long-covid) and the effect on mental-health from COVID-19 intervention measures. However, the choice of measuring the impact of COVID-19 through mortality is consistent with the COVID-19 vaccine prioritization strategies and is generally considered to be the most reliable indicator available. Although better/more timely data (e.g., hospitalizations, ICU admissions) are available in some countries, which allow for different burden of disease characterizations, such data were not available consistently to WHO across all countries.

We introduce mathematical notation to demonstrate the methodology for the first stage of the Global Situational Alert System (GSAS) process, the epidemiological dynamics algorithm. Considering a specific country, let y_t for that country be the "true" number of COVID-19 associated deaths whose exact **time of death** (TOD) falls in the time interval t. For the GSAS process we will consider the interval to be one day, because this is how COVID-19 data are collected and compiled at WHO [1]. Since the reported deaths time series for most countries are not indexed by the event TOD, but instead the time of reporting to WHO (TOR), we will instead use the TOR time-series. Let T denote the most recent time point in the available data.

Since COVID-19 deaths lag cases, the observed number of deaths at time T are determined by the number of cases which happened about 3 weeks before.[1] Hence, in order to make the target metric more timely we consider a **short-term forecast** of mortality. We are able to predict the true number of COVID-19 related mortalities from now and 5 weeks into the future ($7 \times 5 = 35$ days), i.e., we have $\hat{y}_{T+1,T}$, ..., $\hat{y}_{T+35,T}$, where we have used the subscript notation to indicate that the prediction is made given the information available at time T. The choice of a 5-week forecast is discussed at the end of this Appendix. Hence, the predicted total number of COVID-19 associated deaths in a given country within the next 5 weeks per 1 million population will be:

$$\hat{z}_T = \frac{\hat{y}_{T+1,T} + \cdots + \hat{y}_{T+35,T}}{\text{Population}} \times 10^6$$

[1] In practice we use a daily time scale for the time series, but with input data for a given day being smoothed by a 7-day running mean filter.

We define this quantity as the metric of interest for the situational alert classification. We will obtain the different risk classes of the dynamics classification for a country by thresholding $\hat{z}_t$ into the 5 risk categories as follows:

$$\text{RiskClass}_t = \begin{cases} \text{Minimal} & \text{if } \hat{z}_t < h_1 \\ \text{Low} & \text{if } h_1 \leq \hat{z}_t < h_2 \\ \text{Medium} & \text{if } h_2 \leq \hat{z}_t < h_3 \\ \text{High} & \text{if } h_3 \leq \hat{z}_t < h_4 \\ \text{Very High} & \text{if } \hat{z}_t \geq h_4 \end{cases}$$

After this general outline of the classification, in what follows, we discuss the four sub-components of the system:

1. Relationship between the case time series and the deaths time series
2. Case prediction model to get short-term forecasts for the expected reported mortality 35 days into the future
3. Determining the adjustment factor for the reported deaths to bring the projected mortality for a country on a meaningful scale
4. Determine the initial dynamics alert class by thresholding the adjusted projected mortality

1.1.1. Relationship between the cases and deaths time series

Because reported deaths (by TOR) lag reported cases (by TOR), we use the time series of reported cases as a short-term predictor. Since only a certain proportion of reported cases become COVID-19 associated deaths we would need to know:

1. The current case fatality ratio (CFR)
2. The delay distribution between a case appearing in the TOR case time series and the same case appearing TOR mortality time series

Let $\tilde{x}_t$ denote the number of reported cases with TOR at WHO during day t, $t = 1, \ldots, T$. The natural disease progression is infection → positive test → death, where the first transition only happens to cases which are actually tested, and the last transition applies only to cases that die. We therefore consider the following relationship between the time series $\tilde{x}_t$ of reported cases and the time series $\tilde{y}_t$ of reported deaths (each by TOR):

$$\mu_t = E(\tilde{y}_t) = \sum_{s=1}^{\infty} w_s \times \text{CFR}_{t-s} \times \tilde{x}_{t-s}$$

with $0 \leq w_s \leq 1$ being appropriately scaled weights. Such a model can be motivated as follows. Consider a case reported at day t. For cases who die, the fraction being CFR_t, let D denote the number of days between the day of report of the case and the day of report of their death. The support of D is on $1,2,\ldots,D_{\max}$. To make the inference simpler we use a discretized version of $D \sim \text{LogN}(\mu_D, \sigma_D^2)$ and right-truncated at $D_{\max}$ equal to some appropriate quantile of the underlying log-normal distribution (currently the 95% quantile).

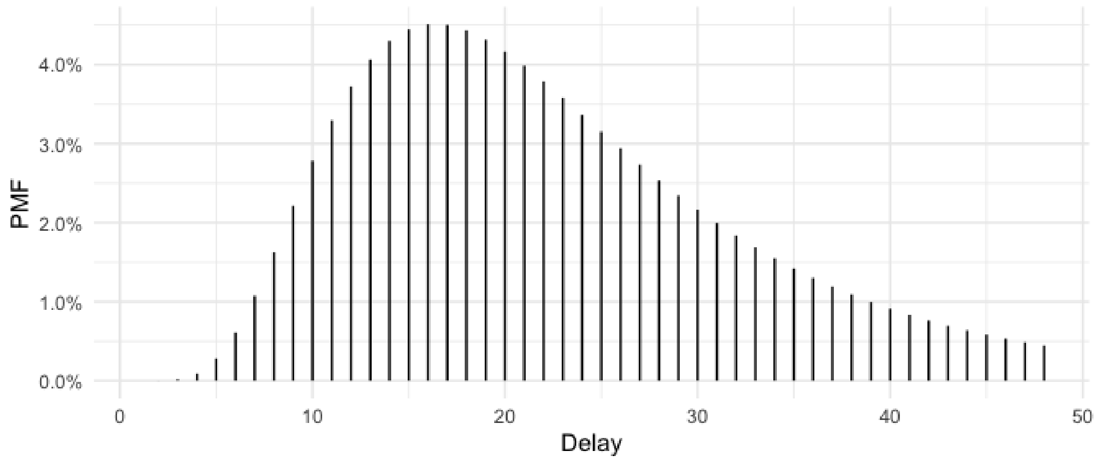

Figure S1 Example probability mass function (PMF) obtained from discretizing an underlying "LogN"("log"(21)=3.0,〖0.4〗^2) distribution

The first weight w_1 in the above equation would then correspond to the PMF at 1, i.e., $P(D = 1)$, and $\text{CFR}_{t-1}w_1$ thus corresponds to the contribution that cases reported at time $t-1$ has on the expected number of reported deaths at time t. Similarly, the 2nd weight together with the CFR at time CFR_{t-2} reflects the contribution of cases reported at time $t-2$ and so on. Using the above model (Figure S1) on a hypothetical case curve with a CFR=25% (note: this high value was chosen for visual purposes in the resulting graph) and the PMF results in the expectation of the deaths time series shown in Figure S2. A total of 8033 observed cases results in 2001 expected deaths.

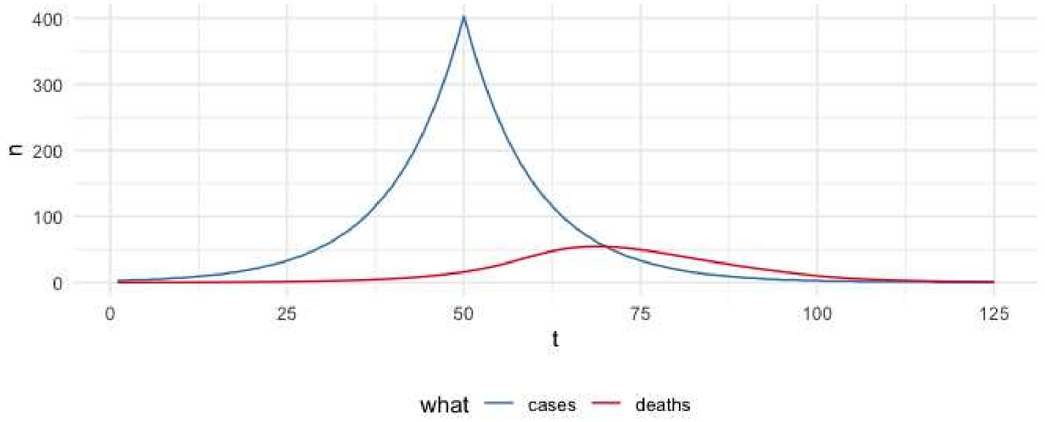

Figure S2 Expectation of the deaths time series from a hypothetical case curve with a CFR=25%

1.1.2 Estimation

Let $\theta = (\text{CFR}, \mu_D, \sigma_D)'$ contain the three parameters of the model to convert between cases and expected deaths, and let σ relate to the variance of an observational model for the time series of reported deaths, i.e.,

$$\tilde{y}_t \sim N(\mu_t(\theta), \sigma^2).$$

Let $W = [t_{\min}, t_{\max}]$ denote a time-window for the deaths time series to use for the fitting. Typically, $t_{\max} = T$ and $t_{\min} = T - \Delta + 1$, where Δ is the window length, e.g., 28 days. We obtain estimates for $(\theta', \sigma)'$ by minimizing the least square deviation between the projection and the observed value of the deaths time series in W. That is, we want to minimize:

$$\sum_{t \in W} \frac{1}{\sigma^2} (\tilde{y}_t - \hat{\tilde{y}}_t(\theta))^2.$$

For the above example time series and letting $\sigma \equiv 1$ we obtain the estimated values shown in Table S1. Knowing the true parameters, we can see that the estimation works well. This is also reflected by the model fit illustrated in Figure S3.

Table S1 Estimated values of (CFR, mu_D, sigma_D) with true values (0.25, 3.00, 0.40)

cfr	mu_D	sigma_D
0.237	3.007	0.457

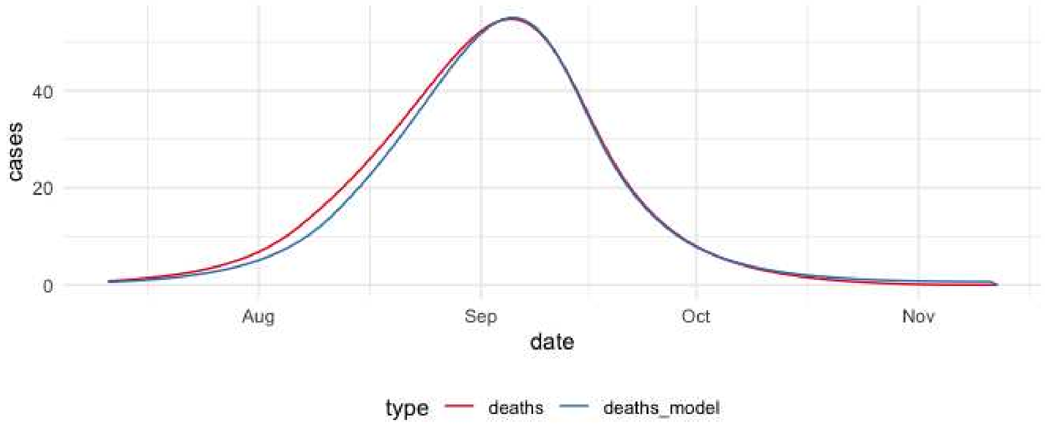

Figure S3 Model fit for death time series where true CFR=25%, mu = 3, sigma = 0.4

1.1.3 Prediction model for cases

The above relationship is driven by disease characteristics of COVID-19, but also the reporting processes of cases and deaths. In order to compute z_t from the above formula, we would also need the observed cases for the future times $T+1, \ldots, T+35$. Here we would use a simple exponential growth model providing predictions based on the observed values of the last 2 weeks:

$$\log(\tilde{x}_t) = \beta_0 + \beta_1 \times t + \epsilon_t = \tilde{\mu}_t^x + \epsilon_t, \quad t = T, \dots T - 13.$$

where $\epsilon_t \overset{\text{iid}}{\sim} N(0, \phi^2)$. This model can then be used to get the predictions $\hat{\tilde{x}}_{t,T}$ for $t = T + 1, \dots T + 35$. Due to the log-transform the model for $\tilde{x}_t$ will be LogN$(\tilde{\mu}_t^x, \phi^2)$. Note: In terms of uncertainty, it is important to distinguish between getting a confidence interval for $\tilde{\mu}_t^x$ and a prediction interval for $\tilde{x}_t$.

1.1.4 Adjustment factor

We assume the following relationship between true COVID-19 associated mortalities y_t and reported mortalities $\tilde{y}_t$ for a given time period t:

$$\tilde{y}_t = c_t \cdot y_t,$$

where $0 \leq c_t \leq 1$ is the **adjustment factor** of COVID-19 associated deaths for the country. One of the big challenges of the dynamics algorithm is a reliable and real-time estimation of c_t.

We further use adjustment factors relating reported COVID-19 deaths to excess mortality.[2] We denote the monthly excess mortality estimates for country k as m_{jk} with $j \in \{1, \dots, 18\}$, where $j = 1$ refers to January 2020, and $j = 18$ to June 2021. Using only estimates of excess mortality from 2021, we obtain the cumulative excess mortality estimate for a given country k for the first six months of 2021 as $M_k = \sum_{j=13}^{18} m_{jk}$. Letting the cumulative reported deaths in in the same time period be $\tilde{Y}_k = \sum_{j=13}^{18} \tilde{y}_j$, we can obtain a country-specific time-constant adjustment factor as $c = \tilde{Y}_k / M_k$.

As country-level adjustment factors may be considered too sensitive we group countries in to four categories according to the World Bank income groups: High-Income Countries (HIC); Upper-Middle Income Countries (UMIC), Lower-Middle Income Countries (LMIC) and Low-Income Countries (LIC). We then use the median adjustment factor within each group as the point estimate c and obtain a 95% confidence interval for the factor using the within group normalized median absolute deviation (NMAD) (Table S2). This corresponds to an estimation of mean and standard deviation in each group, which is robust against outlying observations within each group. The derived mean and standard deviation in the group is then used to formulate a distribution reflecting the uncertainty about the knowledge of the specific adjustment factor of a country. This uncertainty of the adjustment will then be incorporated into the final projection.

Table S2 Estimated adjustment factors grouped by Income groups: High-Income Countries (HIC), Upper-Middle Income Countries (UMC), Lower-Middle Income Countries (LMIC), Low-Income Countries (LIC)

World Bank Group	c	1/c
HIC	0.99	1.01
UMIC	0.54	1.87
LMIC	0.18	5.56
LIC	0.04	22.58

The adjusted mortality projection is then:

$$\hat{z}_T = \frac{\hat{\hat{y}}_{T+1,T} + \cdots + \hat{\hat{y}}_{T+35,T}}{c \times \text{Population}} \times 10^6.$$

1.1.5 Thresholds

We used the 14-day ECDC reporting thresholds scaled to our 35-day period: $h_1 = 35$, $h_2 = 100$, $h_3 = 250$, $h_4 = 500$ deaths per million population for the 35-day period.[3] An alternative representation of these values is $h_1 = 1.00$, $h_2 = 2.86$, $h_3 = 7.14$, $h_4 = 14.29$, deaths per million population per day. Note that we reduce the ECDC 35-day threshold for h_1 from 50 to 35 to reflect the WHO GSAS alert levels.

1.1.6 Data Caveats

The description so far has been based on the ideal situation that data on the cases and deaths time series are readily available. However, in practical operation there can be several issues in the data reporting complicating the analysis.

One issue is to detect possible lack-of-reporting in the two data streams. With the current infrastructure cases or deaths for a given day can be zero because of lack of reporting. We use a simple ad-hoc approach to detect such instances and in response extend the windows used for estimation of, e.g., the case projection model. Furthermore, robust linear regression is used to fit the projection model in order to obtain an outlier robust estimation.[4]

1.2. Examples

In what follows we illustrate the above methodology using two countries with data up to 2021-11-12, which is located in ISO week 45 in 2021.

1.2.1. Romania

1.2.1.1. CFR and Delay

The most recent CFR estimate, i.e., with data up to T=2021-11-12, is 3.0%. The mean waiting time between a COVID-19 associated death appearing in the reported cases time series and

then appearing as a reported death in the deaths time series is 11.6 days (SD: 1.2). The model fit for the sliding window of the death time series used to fit the model is shown in Figure S4.

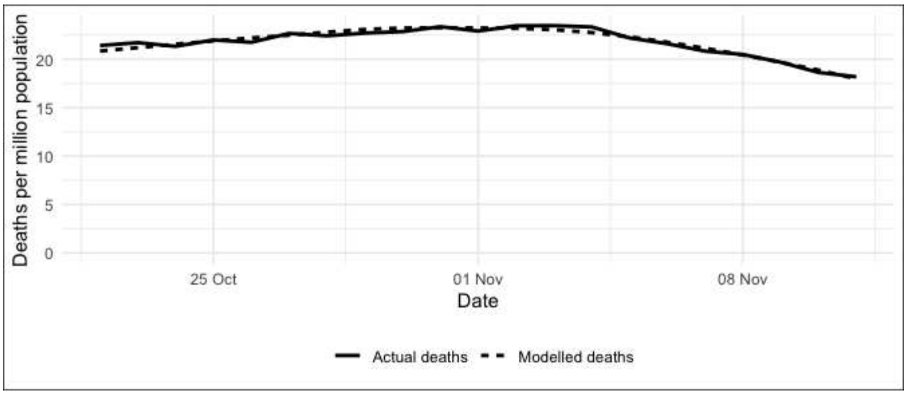

Figure S4 Model fit for the sliding window of the death time series used to fit the model for Romania with data up to T=2021-11-12

1.2.1.2. Case and Death Projections

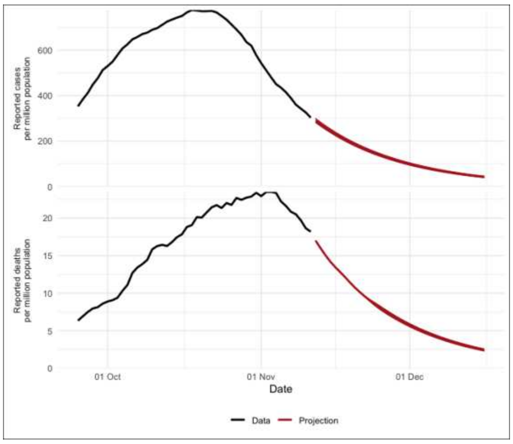

Figure S5 Case and death projections over a 35-day window for Romania with data up to T=2021-11-12 and CFR estimate 3.0%

Summary

- The number of estimated deaths in the next 5 weeks is: 263.5 per 1 million population (95% CI: 259.1 - 268.0).
- The selected adjustment factor is 1.01.
- This means that the adjusted predicted number of deaths in the next 5 weeks is 267.1 (95% CI: 262.7 - 271.7) per 1 million population. Converted to a daily average this corresponds to 7.6 (95% CI: 7.5 - 7.8) deaths per 1 million population per day.

- The alert level class with highest density is **High**.
- The classification after contextual assessment in that week was **Very High**.

1.2.2. Democratic Republic of the Congo

1.2.2.1. CFR and Delay

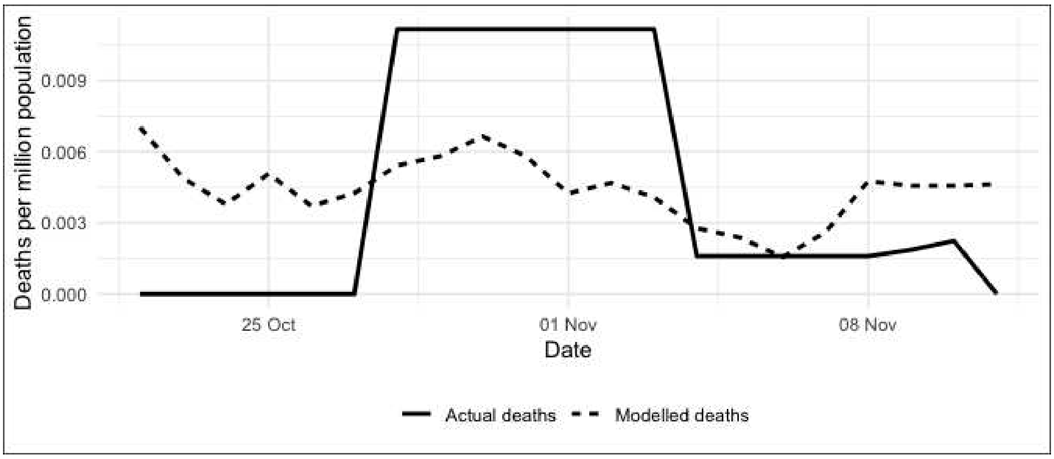

Figure S6 Model fit for the sliding death window for the Democratic Republic of the Congo using data up to T=2021-11-12

The CFR estimate with data up to T=2021-11-12, i.e., the most recent estimate, is 2.6%. The mean waiting time between a COVID-19 associated death appearing in the reported cases time series and then appearing as a reported death in the deaths time series is 10.7 days (SD: 0.5). The model fit for the sliding window of the death time series used to fit the model is shown in Figure S6.

1.2.2.2. Case and Death Projections

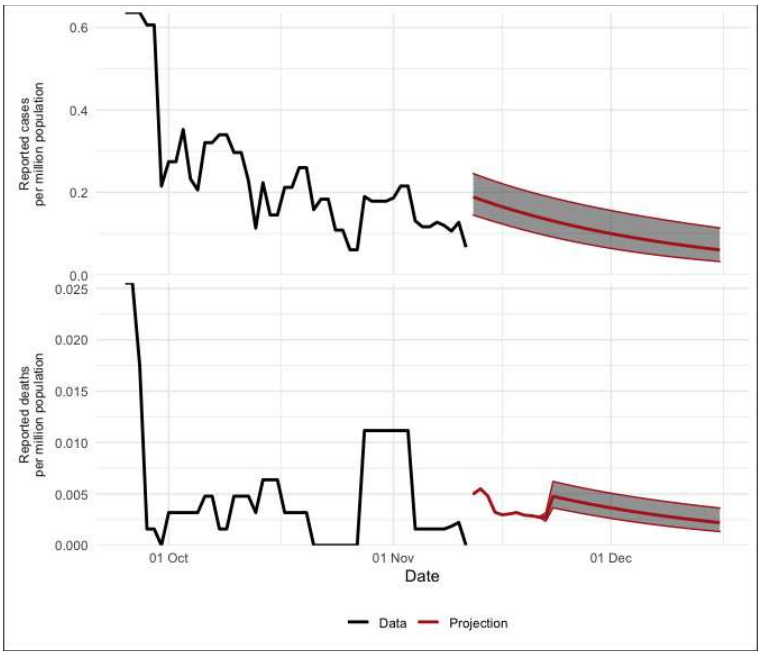

Figure 7 Case and death projections over a 35-day window for Democratic Republic of the Congo with data up to T=2021-11-12 and CFR estimate 2.6%

Summary

- The number of estimated deaths in the next 5 weeks is: 0.1 per 1 million population (95% CI: 0.1 - 0.2).
- The selected adjustment factor is 22.58.
- This means that the adjusted predicted number of deaths in the next 5 weeks is 2.7 (95% CI: 2.1 - 3.5) per 1 million population. Converted to a daily average this corresponds to 0.1 (95% CI: 0.1 - 0.1) deaths per 1 million population per day.
- The alert level class with highest density is **Minimal**.
- Note: After contextual assessment, the classification in that week was **High**.

The example shows the weighting of reported epidemiological data, contextual information, and communicability of results.

1.3. Discussion

By using mortality within the next 5 weeks as our proxy for disease burden, we perform an implicit weighting of current absolute level and current trend. If cases are increasing but the absolute level is low then, due to exponential growth, the overall number in 5 weeks might be large. Likewise, if there is a moderate decreasing trend at a large absolute level, this would still result in a large sum over the 5 weeks. Had we instead chosen a longer window than 5 weeks,

the effect of trend would become more important than the absolute level. However, it would also mean that uncertainty in the projection would increase beyond what can be captured by statistical model uncertainty (e.g., due to behavioral changes). As such, the 5-week projection is a trade-off between statistical robustness and operational needs, where our simple exponential model is about as far as the case projection can be robustly made.

The adjustment factors and thresholding of the algorithm can be seen as one joint step focusing on deaths, which is a straightforward measure of disease burden. A key caveat however is an appropriate adjustment of the reported deaths. In the application we have used an under-reporting inspired approach, which is to be interpreted as an attempt to get approximate evidence-based thresholds, rather than an actual attempt to quantify under-reporting. The associated uncertainty intervals reflect this fact.

References

1 Linton NM, Kobayashi T, Yang Y, *et al.* Incubation Period and Other Epidemiological Characteristics of 2019 Novel Coronavirus Infections with Right Truncation: A Statistical Analysis of Publicly Available Case Data. *J Clin Med* 2020;**9**. doi:10.3390/jcm9020538

2 Msemburi W, Karlinsky A, Knutson V, *et al.* The WHO estimates of excess mortality associated with the COVID-19 pandemic. *Nature* 2023;**613**:130–7. doi:10.1038/s41586-022-05522-2

3 European Centre for Disease Prevention and Control (ECDC). Assessing SARS-CoV-2 circulation, variants of concern, non-pharmaceutical interventions and vaccine rollout in the EU/EEA, 15th update. 2021. https://www.ecdc.europa.eu/sites/default/files/documents/RRA-15th-update-June%202021.pdf (accessed 17 Jan 2023).

4 Koller M, Stahel WA. Nonsingular subsampling for regression S estimators with categorical predictors. *Comput Stat* 2017;**32**:631–46. doi:10.1007/s00180-016-0679-x
