# Supplementary material for: WHO Global Situational Alert System: a mixed methods multistage approach to identify country-level COVID-19 alerts
Source: BMJ Glob Health. 2023 Jul 26;8(7):e012241. doi: 10.1136/bmjgh-2023-012241 (PMC10373705; doi:10.1136/bmjgh-2023-012241)
Supplement: Supplementary data [file bmjgh-2023-012241supp003.pdf]

WHO Global Situational Alert System: a mixed methods multistage approach to identify country-level COVID-19 alerts

Appendix 3:

Data sources

Summary

- Daily cases and deaths count from the member state countries are compiled by the different WHO regional offices and further compiled and verified in WHO-HQ data repository. Those data are transmitted to the WHO-HQ through APIs and the process of fetching the data was automated using XMART on the agreed cut-off times with the respective WHO regions. While the frequency of data reporting is daily for most of the member states, some countries report on certain intervals. The incidence data for the WHO situational alert system is hence calculated using the full epidemiological week (Monday to Sunday) to overcome the reporting bias that could arise from the irregular reporting and some low reporting over the weekends.
- Vaccination coverage was provided by Our World in Data who update their database daily and refers to those who have completed the primary series.
- The Public Health and Social Measures (PHSM) data were obtained from the WHO PHSM Severity Index, calculated from a database of collated information from trackers produced by WHO and the London School of Hygiene and Tropical Medicine and updated on a weekly basis.
- The countries defined as fragile, conflict-affected, and vulnerable (FCVs) were obtained from a UN list which is updated according to the current situation.
- Information for the contextual assessment was obtained from a variety of sources.
    - The WHO Epidemic Intelligence from Open Sources (EIOS) is a dashboard which uses artificial intelligence to select media articles according to categories specified and is updated every two hours. For the WHO situational alert system, a board was created to enable the detection of media articles globally based on categories specific to COVID-19.
    - Search engines such as Google were also used to obtain media articles
    - Further information obtained from WHO regional office COVID-19 situational reports shared with HQ.
    - Specific healthcare information such as the numbers of patients hospitalized was sometimes made available publicly via Ministry of Health websites or Facebook pages and details on SARS-CoV-2 variants, from the ‘variant tracker’ produced by WHO HQ to monitor the presence of variants by country in real-time (Table S2).

Table S2 Data sources used for the WHO situational alert system indicators (December 2021 onwards).

Indicator	Category	Source	Frequency of updates
**Country population figures**	Dynamics (automated)	UN population figures[9]	-
**Incidence of cases**	Dynamics (automated)	WHO Member States[1]	Daily (Monday to Friday)
**Incidence of deaths**	Dynamics (automated)	WHO Member States[1]	Daily (Monday to Friday)
**Vaccination coverage**	Contextual (automated)	Our World in Data	Daily
**Public Health and Social Measures (PHSM)**	Contextual (automated)	WHO PHSM index data[10–12a]	Weekly
**Countries affected by humanitarian emergencies**	Contextual (automated)	WHO countries with Humanitarian Response Plans[8]	As required
**Evidence of the health system struggling with demand (HSP)**	Contextual (manually input)	Epidemic Intelligence from Open Sources (EIOS)[6]	Every two hours
**Impact of other concerning epidemiological signals (ES)**	Contextual (manually input)	Internet search engines e.g., Google [b]	Real-time
**Impact of factors affecting response (TR)**	Contextual (manually input)	WHO Country and Regional Office situation reports	Various (normally at least weekly)
		Ministry of Health websites/Official Facebook pages	Various (depending on frequency of MoH updates)
		WHO variant tracking database (internal)	Various

[a] WHO HQ index used for countries in African region, Eastern Mediterranean region, Region of the Americas, and Western Pacific regions, WHO European regional index used for European region and WHO South-East Asian index used for South-East Asian region. [b] Typically used if information was not available on EIOS for a particular country
