# Supplementary material for: WHO Global Situational Alert System: a mixed methods multistage approach to identify country-level COVID-19 alerts
Source: BMJ Glob Health. 2023 Jul 26;8(7):e012241. doi: 10.1136/bmjgh-2023-012241 (PMC10373705; doi:10.1136/bmjgh-2023-012241)
Supplement: Supplementary data [file bmjgh-2023-012241supp004.pdf]

WHO Global Situational Alert System: a mixed methods multistage approach to identify country-level COVID-19 alerts

Appendix 4: Context methods framework

1. Overview

1.1 Aim

The WHO situational alert framework offers a flexible structure which accounts for the large heterogeneity in data availability and data quality across countries and over time. Countries with more surveillance data available can use this in the decision-making process, whilst those with less data available or data from unverifiable sources can also conduct the assessment with transparency.

1.2 Process summary

The format for generating situational alert classes is to first generate a 'dynamics' classification based on an algorithm using the time series of reported incidence and deaths. The role of the contextual assessment is to additionally inform this statistical decision by adding contextual information. Technically, the decision is to upgrade, maintain or downgrade the dynamics classification based on additional epidemiological and contextual information.

The contextual information considered is partitioned into six indicators – three of which are pre-populated using data (see Table 2.1) and the other three of which are manually updated (see Table 2.2). Users assessing a given country would select a level at which an indicator is present (if known) and an associated level of trust in the information/source this choice is based on. In the first instance, the default level assumes the factor is not present with 'low' trust. However, in future assessments, the defaults will be country-specific by assuming the level and trust inputs from the previous weeks assessment.

Based on these choices, a 'recommendation' is automatically generated for users. Users may choose to accept the recommendation, in which case no further input is necessary; or may choose to override the recommendation due to expert knowledge not captured by the indicators, which can be documented accordingly. For instance, a country may need to be upgraded two or more levels in the presence of an acute event where data collection is impacted. The final classification possibilities remain as follows: 'No Data', 'Minimal', 'Low', 'Medium', 'High', Very High' and 'Critical'.

2. Methods

2.1 Methods summary

The revised context assessment framework is based on a point-based system for a set of selected indicators. Each level of a particular indicator is assigned points in terms of the weight of evidence it provides for a potentially bad COVID-19 situation in the country within the coming weeks. The assigned points are then summed up to determine an overall score. An upgrade of the dynamics classification would be recommended if the score passes a given threshold. Because the degree of certainty about the different indicators can vary substantially between countries and settings, the framework also takes the level of trust around the different indicators into account.

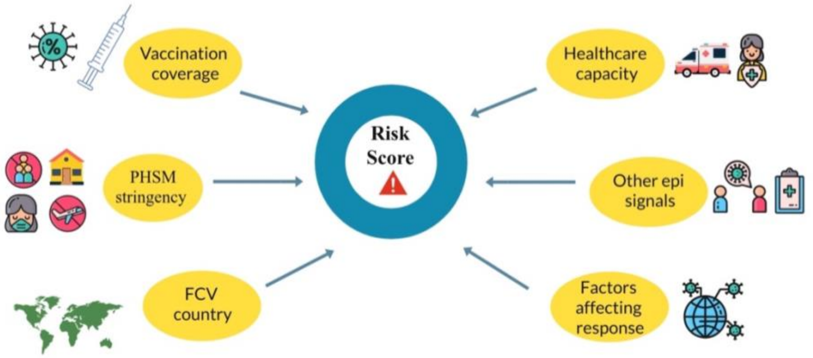

Figure 2.1 Indicators used to form the context risk score where ‘vaccination coverage’, ‘PHSM stringency’, ‘FCV country’ are prepopulated using existing data and ‘healthcare capacity’, ‘other epi signals’ and ‘factors affecting response’ are manually updated

Formally speaking, the score is not represented by a single value, but as a distribution over the different score values in order to take uncertainty in the assessments into account. The variance of this distribution is determined by the trust settings for the six indicators. The system thus computes the probability that the score will exceed the specified upgrade threshold and the probability that the score will fall below the specified downgrade threshold, and advises to downgrade, maintain or upgrade based on which decision has the highest probability.

2.2 Pre-populated indicators

The availability and quality of data varies substantially across countries which are to be assessed. Where data is available, indicator and trust levels will be assigned, which the user may override, if they have more detailed or recent information. Of note, is that the risk associated with PHSM

stringency levels is currently allocated in a predictive manner, where it is assumed that ‘low’ stringency in the event of a large outbreak will likely enable transmission.

Table 2.1 Pre-populated indicators, levels and associated risk scores

Indicator	Levels	Notes
Vaccination coverage (V)	• Very High (> 80%)<br>• High (60 – 80%)<br>• Moderate (40 - 60%)<br>• Low (20 - 40%)<br>• Very Low (< 20%)	• Pre-populated with ‘high’ trust
PHSM stringency levels (PHSM)	• Very High (> 65%)<br>• High (50 - 65%)<br>• Moderate (35 - 50%)<br>• Low (< 35%)	• Pre-populated using WHO PHSM index data<br>• The ‘moderate’ trust level is selected to increase the uncertainty around the assumed PHSM stringency level to account for variation in adherence”
FCV country (FCV)	• No<br>• Yes	• Pre-filled using list of FCV countries<br>• Adds small baseline risk to the final score - acute events within these countries in a given week must still be accounted for in the manually updated ‘TR’ indicator

2.3 Manually updated indicators

The three indicators to be manually updated by the user with available information are shown in Table 2.2, relating to (i) ‘Evidence of health system struggling with demand (HSP)’, (ii) ‘Impact of other concerning epidemiological signals (ES)’ and (iii) ‘Impact of factors affecting transmission or response (TR)’, where all three indicators are assessed in relation to their severity.

It is important to note that the ‘possible signals’ documented in Table 2.2 are only included as an illustration of what type of information is considered within each indicator and is not a list of information that assessors were expected to gather for the assessment. The assessment was conducted with only the available information with signals categorised according to examples shown in Table 2.2.

The initial pilot run assumed that the state of the indicator is ‘absent’ but with ‘low’ trust, unless otherwise stated. In future assessments, the defaults were country-specific by assuming the level and trust inputs from the previous weeks assessment.

Table 2.2 Manually updated indicators, possible signals related to that indicator, indicator levels and associated risk scores. Note that signals are described by assessors in free text columns when completing the weekly evaluation

Indicator	Possible signals	Levels
Evidence of health system struggling with demand (HSP)	• Hospital demand likely to exceed capacity in next 1-2 weeks<br>• Shortages of supplies (e.g., oxygen) or personnel<br>• Mortuaries/burial grounds overwhelmed<br>• Other signals of health system pressure present	• No evidence<br>• Some signs of pressure<br>• Signs of high pressure<br>• System overwhelmed
Impact of other concerning Epidemiological Signals (ES)	• Testing related concerns (e.g., large increase in TPR or change in testing policy)<br>• Concurrent outbreaks<br>• Large changes in variants of concern circulating<br>• Other epidemiological signals increasing risk	• None<br>• Moderate<br>• Significant
Impact of factors affecting transmission or response (TR)	• Instability or insecurity related to acute events<br>• Logistical challenges<br>• Large one-off mass gatherings<br>• Border re-opening or other mass movement<br>• Other factors increasing risk, relating to transmission or response	• None<br>• Moderate<br>• Significant

2.4 Thresholds

An 'upgrade threshold' and a 'downgrade threshold' are predefined, where the system recommends either a downgrade, maintain or upgrade based on which decision class has the highest probability. Initial values were informed by expert opinion and estimation from prospective pilots and the thresholds were reviewed regularly based on feedback from end-users and data-driven evaluation.

2.5 Trust levels

In addition to accounting for the levels of an indicator, the associated trust may also be accounted for through the corresponding trust indicator. This aims to capture uncertainty in the assessment resulting from different amounts of and quality of information available between countries and weeks.

In all cases the contribution to the expected value of the overall score by each indicator is the weighted average (weights according to the selected level of trust) of the possible scores associated with each level of that indicator. Furthermore, the variability of the overall score is composed of the variability of the weights for the indicator – this is largest for the trust setting 'unknown' and zero for the trust level 'high'.

The interpretation of the trust levels, along with recommendations for when each trust level is most appropriate in practice, is described in Table 2.3. To make this clearer, a worked example is presented in Section 4.

Table 2.3 Trust levels in available sources and assessment

Trust level	Interpretation	When to use
High	• Assigns 100% of weight to the level selected – the points assigned to this level are added to the final score.	• Reliable data is available or other event-based information is from a trusted source (e.g., sitreps)
Moderate	• Indicator level chosen is most likely, but situations described by levels either side of the chosen level are also possible	• Some information is available but from a less reliable source (e.g., media outlet)
Low	• Something is known about the situation from evidence or expert knowledge but with very low certainty<br>• The level selected is the most likely but the two neighbouring levels for a given indicator are also possible	• Very limited or no information is available on that indicator<br>• Some information is available, but the source is not trusted
Unknown	• All levels within the indicator are equally likely<br>• An average of the possible scores for that indicator is added to the score	• When no information is consistently output from a country, with limited or no media access, and so the situation is truly unknown by HQ and RO

2.5 Underlying Statistical Method

Single Indicator

Consider a single indicator I of the contextual assessment which has k states. The contextual assessment provides evidence about the states of I by shaping the distribution over $i_1,\ldots,i_k$, which is given by the probability mass function (PMF) over the support of possible points. Say there are two ways to specify evidence:

1. select one of the states or
2. it's not possible to select a state, because the situation is unknown.

Rules: We shall reflect 1. by putting $P(I=i_c)=1$ for the selected class i_c. We shall reflect 2. by setting $P(I=i_j)=1/k$ for $j=1,\ldots,k$, i.e. we use a discrete uniform prior over the indicator states.

Reflection of *trust*: The different levels of trust in the data source modify the variance of the distribution over the states. However, if the distribution is already the discrete uniform, then no additional modification needs to be made. Thus, we only have to consider situation 1., i.e. a specific state was selected and we handle the three trust levels as follows:

- high: no changes of the distribution
- medium: modify the posterior so the neighboring states are also assigned some mass.
- low: modify the posterior even more, i.e., also assigning mass to states further away from the select. Basically, the idea is to get closer to the uniform distribution.

Statistically, we reflect the above choices for the trust by smearing the distribution of I at index i by considering a kernel for the indices $i+\Delta$, where $\Delta=-w,\ldots,0,\ldots,w$ and where w is either 1

(medium trust) or two (low trust). The weights for the kernel are proportional to $s^{|\delta|}$ for $\delta \in \Delta$ and where $0<s<1$ is a scaling factor. Note that we normalize the weights in case any of the indices in $i+\Delta$ fall outside the interval 1,…,k.

Summation

Let $\mathcal{J}=(I_1,\ldots,I_N)$ be the set of indicators to consider. The additive score of the indicators is defined as:

$$Score = \sum_{i=1}^{N} I_i$$

i.e., we sum up the points of all indicators. As long as the support of each indicator only consists of integer scores, the resulting value will again be an integer score. The proposed uncertainty enhancement now consists of deriving the distribution of the score variable. This requires the probability mass function (PMF) of the discrete convolution of the indicators' points.

The distribution over the possible points provided by each indicator can then be used to illustrate the uncertainty in the summation score. Instead of obtaining the PMF of the Score by repeatedly applying the convolution formula for the sum of two independent random variables, one can approximate the convolution by approximation the PMF using only it's mean and variance. By the assumption of independence between the different indicators, this can easily be computed by summation of expectation and variance, respectively, of each indicator:

$$E(Score) = \sum_{i=1}^{N} E(I_i)$$

$$\mathrm{Var}(\langle Score \rangle) = \sum_{i=1}^{N} \mathrm{Var}(I_i)$$

Note that this ignores any occurrence of kurtosis and skewness etc. However, especially if many indicators are summed, this approximation is likely to work well. For computational purposes we use this approximation.

3. Infrastructure

3.1 User interface

The system is implemented as part of an updated Excel spreadsheet framework for the SOC process. Dropdown menus allow users to select the indicator and trust levels (see Figure 3.1).

The user interface contains a 'suggested classification' column and separate 'final classification' column, where the recommendation is automatically generated based on the dynamics classification plus the default selections for the indicator columns, in the first instance. As the indicator columns are updated with available evidence, the recommendation will automatically update based on the selections.

country	HSP_level	HSP_trust	ES_level	ES_trust	TR_level	TR_trust	VAX_level	VAX_trust	PHSM_level	PHSM_trust
Bangladesh	No evidence	low	None	low	None	low	Very Low (< 20%)	high	Moderate (33 - 66%)	moderate
Bhutan	No evidence	low	None	low	None	low	High (60 - 80%)	high	Moderate (33 - 66%)	moderate
Democratic People's Republic of Korea	No evidence	low	None	low	None	low	Moderate (40 - 60%)	high	High (> 66%)	moderate
India	No evidence	low	None	low	None	low	Low (20 - 40%)	high	Moderate (33 - 66%)	moderate
Indonesia	No evidence	low	None	low	None	low	Low (20 - 40%)	high	High (> 66%)	moderate
Maldives	No evidence	low	None	low	None	low	High (60 - 80%)	high	Moderate (33 - 66%)	moderate
Myanmar	No evidence	low	None	low	None	low	Very Low (< 20%)	high	High (> 66%)	moderate
Nepal	No evidence	low	None	low	None	low	Low (20 - 40%)	high	Moderate (33 - 66%)	moderate
Sri Lanka	No evidence	low	None	low	None	low	High (60 - 80%)	high	Moderate (33 - 66%)	moderate
Thailand	No evidence	low	None	low	None	low	Moderate (40 - 60%)	high	Moderate (33 - 66%)	moderate
Timor-Leste	No evidence	low	None	low	None	low	Low (20 - 40%)	high	High (> 66%)	moderate

Figure 3.1 Example infrastructure sheet for the WHO South-East Asian region showing the context indicators and associated trust levels, which produce a ‘regrade decision’ and ‘suggested classification’ which can be either accepted as the final classification or overruled based on expert knowledge

4. Worked example

To demonstrate how to conduct the assessment, we use the example of Madagascar from assessment week 2022-02-21. The available information is shown in Table 4.1, along with the level and trust selected, and the justification for the choices.

As for many other countries in the world, the initial dynamics assessment based on reported cases and deaths generates an alert level of ‘Low’ as COVID-19 cases are declining. Additional contextual information recommends an ‘upgrade,’ from ‘Low’ to ‘Medium,’ however this was further escalated by the global and regional office teams to a ‘Critical’ alert for reasons described in Table 4.1.

Table 4.1 Contextual assessment for Madagascar using available information in week 2022-02-21, where the outcome was to upgrade from ‘Low’ to ‘Medium’ and was further escalated by global and regional teams to ‘Critical’ due to the additional context information

Indicator	Information available	Level	Trust	Reason
**Evidence of health system pressure (HSP)**	Several essential health services disrupted	Signs of High Pressure	Low	Reports of significant healthcare
**Impact of additional concerning epidemiological signals (ES)**	Declining trends of COVID-19 but possible disruption to testing, reporting etc.	Moderate	Moderate	Situation may affect testing and reporting as well as concurrent outbreaks
**Impact of factors affecting transmission or response (TR)**	Cyclone affecting health services and schools. Over 60k people displaced	Significant	High	Potentially significant impact on transmission and response activities
**Vaccination coverage (VAX)**	<1% reported to have received their primary series of vaccination	Very Low (<20%)	High	Low vaccination rates imply further increase in healthcare burden if cases increase

**Public Health and Social Measures index (PHSM)**		Moderate (33 – 66%)	Moderate	PHSM index informs level with moderate trust due to lack of data on adherence
**FCV country (FCV)**		No		Not included on the UN list

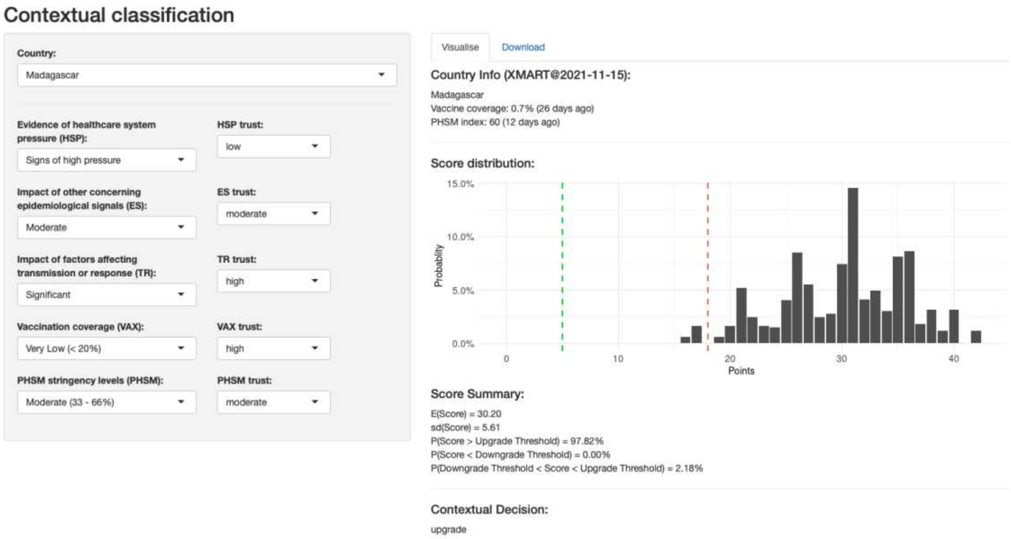

Figure 4.1 Context assessment for Madagascar in assessment week 2022-02-21 where the recommendation to 'upgrade' is in strong agreement with the decision made to upgrade multiple levels by WHO global and regional teams
