# Supplementary material for: WHO Global Situational Alert System: a mixed methods multistage approach to identify country-level COVID-19 alerts
Source: BMJ Glob Health. 2023 Jul 26;8(7):e012241. doi: 10.1136/bmjgh-2023-012241 (PMC10373705; doi:10.1136/bmjgh-2023-012241)
Supplement: Supplementary data [file bmjgh-2023-012241supp005.pdf]

WHO Global Situational Alert System: a mixed methods multistage approach to identify country-level COVID-19 alerts

Appendix 5:

Supplementary results

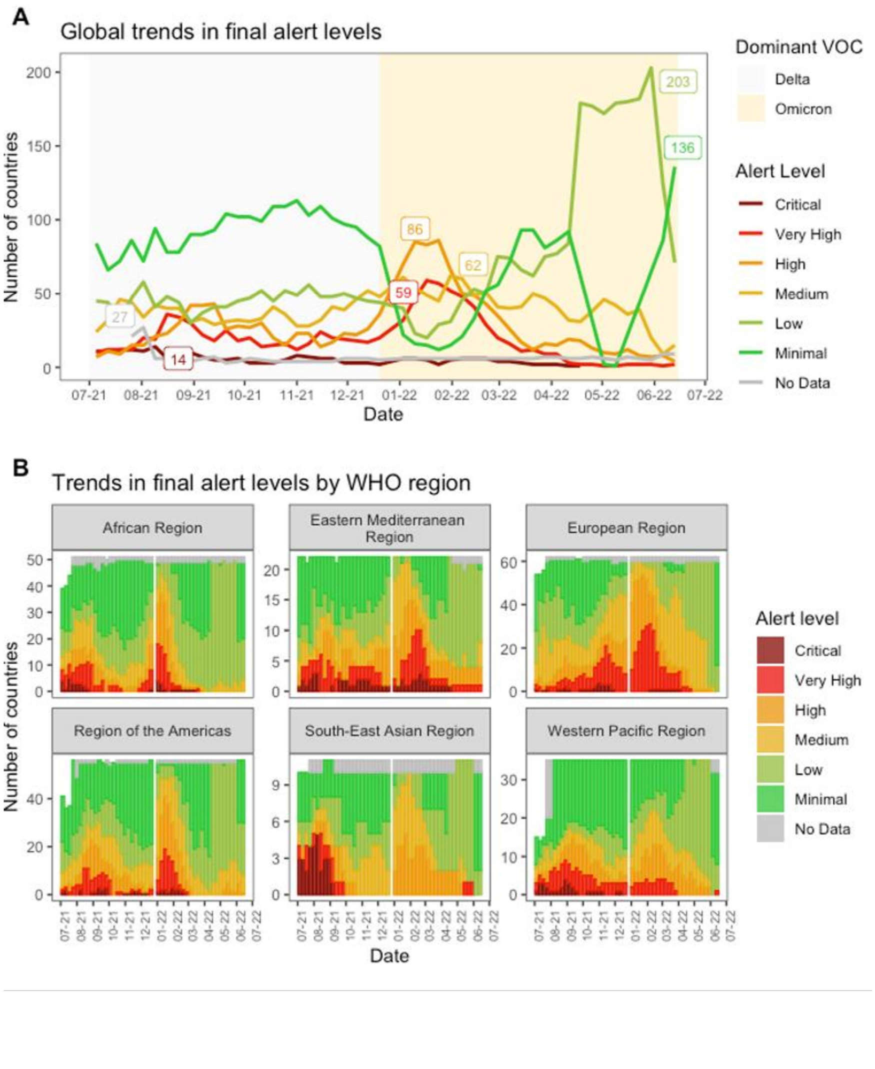

Figure S1 Trends in (A) global and (B) regional final alert levels during Delta and Omicron dominance between July 2021 and June 2022. Notes: (A) the dominant VOC timelines shown in is according to sequences submitted to GISAID; (B) no assessment was performed on the last week of December 2021, as shown by the break in the figure.

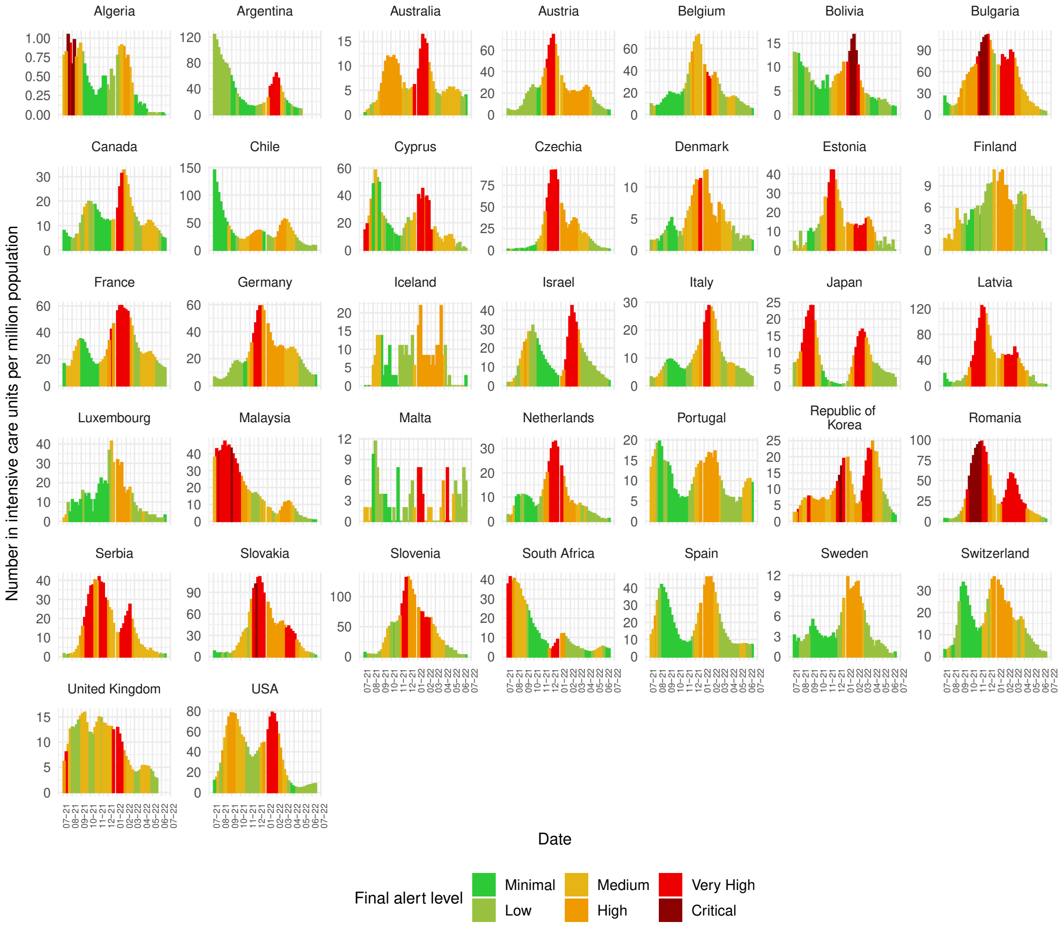

Figure S2: Trends in numbers of cases in ICUs per million population colored by the situational alert level, for a subset of countries in the European region, region of the Americas and Western Pacific region for which this information was available. External data source: Our World in Data. Note that the hospitalization data (availability and quality) varies among WHO member states based on a variety of factors and the data above is not representative of all member states.

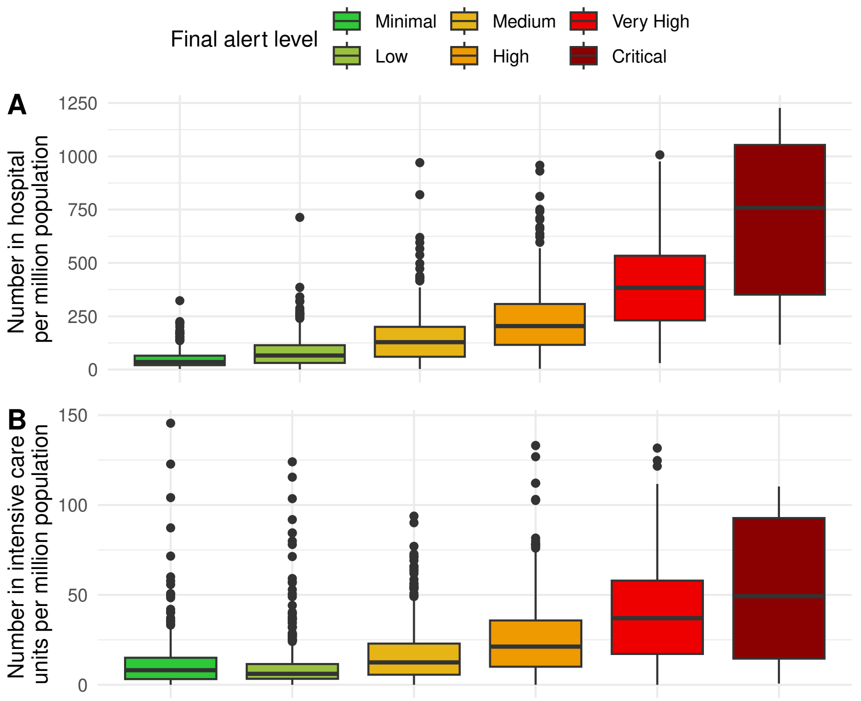

Figure S3: (A) Number of people in hospital per million population and (B) number of people in intensive care units per million across alert levels for the subset of countries in the European region, region of the Americas and Western Pacific region for which this information was available. Note that the hospitalization data (availability and quality) varies among WHO member states based on a variety of factors and the data above is not representative of all member states.
